# Supplementary material for: In situ structural analysis of the Yersinia enterocolitica injectisome
Source: eLife. 2013 Jul 30;2:e00792. doi: 10.7554/eLife.00792 (PMC3728920; doi:10.7554/eLife.00792)
Supplement: Supplementary file 1. [file elife00792s001.docx]

**Supplementary File 1A** – Crystallographic data collection and refinement statistics for YscD^150-362^ and YscD^150-347^ G283P. Due to poor refinement statistics of YscD^150-362^ only coordinates for YscD^150-347^ G283P were deposited at the Protein Data Bank (http://www.pdb.org)

| **Data collection** | **YscD^150-362^** | **YscD^150-347^ G283P** |
| --- | --- | --- |
| Space group | P2_1_ | P2_1_ |
| Cell dimensions |  |  |
| a, b, c (Å) | 48.2, 29.8, 69.9 | 38.1, 51.7, 50.8 |
| α, β, γ (°) | 90.0, 97.1, 90. 0 | 90.0, 106.0, 90.0 |
|  |  |  |
| Resolution (Å) | 29.6-2.7 | 29.8 - 1.4 |
| (highest shell (Å)) | (2.8-2.7) | (1.48 - 1.40) |
| R_merge_ | 9.9 (63.5) | 3.6 (55.2) |
| I/σI | 16.6 (4.4) | 21.0 (2.3) |
| Completeness (%) | 99.2 (98.9) | 99.5 (99.7) |
| Redundancy | 7.2 (7.3) | 3.7 (3.5) |
|  |  |  |
| **Refinement** |  |  |
| R_work_/R_free_ | 29.5/35.3 | 18.9/22.6 |
| B factors |  |  |
| Wilson B (Å^2^) | 47.03 | 22.1 |
| R.m.s deviations |  |  |
| Bond lengths (Å) | 0.006 | 0.009 |
| Bond angles (°) | 1.277 | 1.238 |
| Ramachandran |  |  |
| favored /outliers (%) | 83.9/5.7 | 99.5/0 |
| Molprobity score/percentile | 3.62/15^th^ | 1.40/89^th^ |
| PDB code | - | 4alz |

* Values in parentheses refer to the highest resolution shell

**Supplementary File 1B** – *Y. enterocolitica* strains

| **Strain** | **Relevant characteristics** | **Parent** | **Mutator Plasmid** | **References** |
| --- | --- | --- | --- | --- |
|  |  |  |  |  |
| E40 (WT) | *pYVe40* |  |  | ([Sory *et al.*, 1995](#_ENREF_6)) |
| AD4051 | *∆yscD* | E40 |  | ([Diepold *et al.*, 2010](#_ENREF_2)) |
| IML421 | *yopH_Δ1-352_, yopO_Δ65-55_, yopP_238_, yopE_21_, yopM_23_, yopT_135_ (ΔyopHOPEMT)* |  |  | ([Iriarte & Cornelis, 1998](#_ENREF_3)) |
| IML421asd | *ΔyopHOPEMT* *asd_Δ292-610_^1)^* (biosafety level 1 strain requiring mDAP^2)^ for growth) | IML421 | pMK3 | this work |
| AD4085 | *ΔyopHOPEMT* *asd_Δ292-610_ egfp-yscQ*^+^ | IML421asd | pAD118 | this work |
| AD4086 | *ΔyopHOPEMT* *asd_Δ292-610_ egfp-yscQ*^+^ *yadA_686-956_* | AD4085 | pLJM31 | this work |
| MA4039 | *ΔyopHOPEMT* *asd_Δ292-610_ egfp-yscQ*^+^ *minD* | AD4085 | pMA87 | this work |
| MA4053 | *ΔyopHOPEMT* *asd_Δ292-610_ minD* | IML421asd | pMA87 | this work |
| MA4055 | *ΔyopHOPEMT* *asd_Δ292-610_ egfp-yscQ*^+^ *minD yadA_686-956_* | MA4039 | pLJM31 | this work |
| MA4056 | *ΔyopHOPEMT* *asd_Δ292-610_ minD yadA_686-956_* | MA4053 | pLJM31 | this work |

^1)^ asd = aspartate-β-semialdehyde Dehydrogenase

^2)^ mDAP = meso-diaminopimelic acid

**Supplementary File 1C** – Plasmids

| **Plasmid** | **Relevant characteristics** | **References** |
| --- | --- | --- |
|  |  |  |
| pKNG101 | oriR6K, mobRK2, Sm^R^, sacB (sucrose sensitivity) | ([Kaniga *et al.*, 1991](#_ENREF_4)) |
| pMK3 | pKNG101 *asd_Δ292-610_* | this work |
| pMA87 | pKNG101 *∆minD* | this work |
| pAD118 | pKNG101 *egfp-yscQ*^+^ | ([Diepold et al., 2010](#_ENREF_2)) |
| pLJM31 | pKNG101 *yadA_686-956_*^3)^ | ([Mota *et al.*, 2005](#_ENREF_5)) |
| pRS6 | pACYC184 *cat*::*yscW* | ([Allaoui *et al.*, 1995](#_ENREF_1)) |
| pBAD/mycHisA | pBR322 origin, araBAD promoter, Amp^R^ | Invitrogen |
| pMA6 | pBAD *yscC* | this work |
| pMA11 | pBAD *yscD* | this work |
| pUW22 | pBAD *yscD* G283P | this work |
| pGEX-6P-1 | pBR322 origin, *lacI*, Ptac promotor, Amp^R^ | Invitrogen |
| pAD154 | pGEX *yscD*^150-418^ | this work |
| pUWSS1 | pGEX *yscD*^150-347^ | this work |
| pUWSS2 | pGEX *yscD*^150-362^ | this work |
| pUWSS3 | pGEX *yscD*^150-347^ G283P | this work |

^3)^ Note: this vector inserts itself into the middle of *yadA* and therefore disrupts *yadA*.

**Supplementary File 1D** – List of used oligonucleotides

| **No.** | **Sequence** | **features** | **cloning of** | **template** |
| --- | --- | --- | --- | --- |
| 3541 | GATCGTCGACATGGTCGGCTCAGTA^4)^ | SalI restriction site | pMK3 | genomicDNA E40 |
| 3542 | GATCTCTAGATTCGCAGCATACGGC^4)^ | XbaI restriction site | pMK3 | genomicDNA E40 |
| 3543 | CAGTGAATTCCGGCGTCAATCCAATA^4)^ | EcoRI restriction site | pMK3 | genomicDNA E40 |
| 3544 | GACTGAATTCGTGACTGCGGCCACT^4)^ | EcoRI restriction site | pMK3 | genomicDNA E40 |
| 6416 | CATGGTCGACATTGCCGACGGCAATATTC^4)^ | SalI restriction site | pMA87 | genomicDNA E40 |
| 6417 | AAAGTCTAACAAAGCCATGGTGAAATGGATTCCTTGTCAAAAG | overlap to 6418 | pMA87 | genomicDNA E40 |
| 6418 | CTTTTGACAAGGAATCCATTTCACCATGGCTTTGTTAGACTTT | overlap to 6417 | pMA87 | genomicDNA E40 |
| 6419 | CATGTCTAGACCGGTAATGTCACGTTAAG^4)^ | XbaI restriction site | pMA87 | genomicDNA E40 |
| 5013 | GACTCCATGGCTTTTCCGCTACATTCTTTTTTC^4)^ | NcoI restriction site | pMA6 | pYVe40 |
| 5014 | CTGAATTCACAATACGCCACGCTTAGGTGC^4)^ | EcoRI restriction site | pMA6 | pYVe40 |
| 5014 | CTGAATTCACAATACGCCACGCTTAGGTGC^4)^ | EcoRI restriction site | pMA6 | pYVe40 |
| 5084 | ACTGCAACATGTCTTGGGTCTGTCGTTTTTATCAAGG^4)^ | PciI restriction site | pMA11/pUW22 | pYVe40 |
| 5085 | ATCGAAGCTTCATCGAGGTTTACCTCCATTGAGC^4)^ | HindIII restriction site | pMA11/pUW22 | pYVe40 |
| 5174 | GACTGGATCCAACCAGGATGGACAACTTGTT^4)^ | BamHI restriction site | pAD154 | pYVe40 |
| 5175 | GACTCTCGAGAATTGTGTCATCGAGGTTTACC^4)^ | XhoI restriction site | pAD154 | pYVe40 |
| 6939 | GAAAATAAAGTGCGGATTGCC**CCT**AATCAACGCAAGCGGCTTGATGC^5)^ | G283P mutation | pUW22 | pYVe40 |
| 6940 | GCATCAAGCCGCTTGCGTTGATT**AGG**GGCAATCCGCACTTTATTTTC^5)^ | G283P mutation | pUW22 | pYVe40 |
| UWSS1-fwd | CTAAATTAGAACTGGTCAATGTC**TGA**GGGCAACCCCAGCATGATG^5)^ | G348STOP mutation | pUWSS1 | pAD154 |
| UWSS1-rev | CATCATGCTGGGGTTGCCC**TCA**GACATTGACCAGTTCTAATTTAG^5)^ | G348STOP mutation | pUWSS1 | pAD154 |
| UWSS2-fwd | GAATTTTGAGGTGCAA**TAG**ATCTCGTTAGGGAAAG^5)^ | A363STOP mutation | pUWSS2 | pAD154 |
| UWSS2-rev | CTTTCCCTAACGAGAT**CTA**TTGCACCTCAAAATTC^5)^ | A363STOP mutation | pUWSS2 | pAD154 |
| UWSS3-fwd | GATTGCC**CCG**AATCAACGCAAGCGGCTTGATGCATTAC^5)^ | G283P mutation, G348STOP mutation | pUWSS3 | pUWSS1 |
| UWSS3-rev | GCGTTGATT**CGG** GGCAATCCGCACTTTATTTTCTACACC^5)^ | G283P mutation, G348STOP mutation | pUWSS3 | pUWSS1 |

^4)^ Restriction sites are underlined

^5)^ Mutated codons are bold

Allaoui, A., R. Scheen, C. Lambert de Rouvroit & G. R. Cornelis, (1995) VirG, a Yersinia enterocolitica lipoprotein involved in Ca2+ dependency, is related to exsB of Pseudomonas aeruginosa. *J. Bacteriol* **177**: 4230-4237.

Diepold, A., M. Amstutz, S. Abel, I. Sorg, U. Jenal & G. R. Cornelis, (2010) Deciphering the assembly of the Yersinia type III secretion injectisome. *EMBO J.* **29**: 1928-1940.

Iriarte, M. & G. R. Cornelis, (1998) YopT, a new Yersinia Yop effector protein, affects the cytoskeleton of host cells. *Mol. Microbiol.* **29**: 915-929.

Kaniga, K., I. Delor & G. R. Cornelis, (1991) A wide-host-range suicide vector for improving reverse genetics in gram-negative bacteria: inactivation of the blaA gene of Yersinia enterocolitica. *Gene* **109**: 137-141.

Mota, L. J., L. Journet, I. Sorg, C. Agrain & G. R. Cornelis, (2005) Bacterial injectisomes: needle length does matter. *Science* **307**: 1278.

Sory, M. P., A. Boland, I. Lambermont & G. R. Cornelis, (1995) Identification of the YopE and YopH domains required for secretion and internalization into the cytosol of macrophages, using the cyaA gene fusion approach. *Proc. Natl. Acad. Sci. USA.* **92**: 11998-12002.
